# Association of in vivo retention of [^18^f] flortaucipir pet with tau neuropathology in corresponding brain regions

**DOI:** 10.1007/s00401-024-02801-2

**Published:** 2024-09-19

**Authors:** Tove Freiburghaus, Daria Pawlik, Kevin Oliveira Hauer, Rik Ossenkoppele, Olof Strandberg, Antoine Leuzy, Jonathan Rittmo, Cécilia Tremblay, Geidy E. Serrano, Michael J. Pontecorvo, Thomas G. Beach, Ruben Smith, Oskar Hansson

**Affiliations:** 1 Clinical Memory Research Unit, Department of Clinical Sciences Malmö, Faculty of Medicine, Lund University, Lund, Sweden; 2 Department of Neurology, Skåne University Hospital, Lund, Sweden; 3 Banner Sun Health Research Institute, Sun City, AZ USA; 4 Amsterdam Neuroscience, Neurodegeneration, 1081 HV Amsterdam, The Netherlands; 5 Alzheimer Center Amsterdam, Neurology, Vrije Universiteit Amsterdam, Amsterdam UMC Location VUmc, 1081 HZ Amsterdam, The Netherlands; 6 Avid Radiopharmaceuticals, Philadelphia, PA USA; 7 Memory Clinic, Skåne University Hospital, 20502 Malmö, Sweden

**Keywords:** Flortaucipir, AT8, Neuropathology, Tau, PET, Alzheimer’s disease

## Abstract

[^18^F]Flortaucipir is an FDA-approved tau-PET tracer that is increasingly utilized in clinical settings for the diagnosis of Alzheimer’s disease. Still, a large-scale comparison of the in vivo PET uptake to quantitative *post-mortem* tau pathology and to other co-pathologies is lacking. Here, we examined the correlation between in vivo [^18^F]flortaucipir PET uptake and quantitative *post-mortem* tau pathology in corresponding brain regions from the AVID A16 end-of-life study (*n* = 63). All participants underwent [^18^F]flortaucipir PET scans prior to death, followed by a detailed *post-mortem* neuropathological examination using AT8 (tau) immunohistochemistry. Correlations between [^18^F]flortaucipir standardized uptake value ratios (SUVRs) and AT8 immunohistochemistry were assessed across 18 regions-of-interest (ROIs). To assess [^18^F]flortaucipir specificity and level of detection for tau pathology, correlations between [^18^F]flortaucipir SUVR and neuritic plaque score and TDP-43 stage were also computed and retention was further assessed in individuals with possible primary age-related tauopathy (PART), defined as Thal phase ≤ 2 and Braak stage I–IV. We found modest-to-strong correlations between in vivo [^18^F]flortaucipir SUVR and *post-mortem* tau pathology density in corresponding brain regions in all neocortical regions analyzed (rho-range = 0.61–0.79, p < 0.0001 for all). The detection threshold of [^18^F]flortaucipir PET was determined to be 0.85% of surface area affected by tau pathology in a temporal meta-ROI, and 0.15% in a larger cortical meta-ROI. No significant associations were found between [^18^F]flortaucipir SUVRs and *post-mortem* tau pathology in individuals with possible PART. Further, there was no correlation observed between [^18^F]flortaucipir and level of amyloid-β neuritic plaque load (rho-range =  – 0.16–0.12; *p* = 0.48–0.61) or TDP-43 stage (rho-range =  – 0.10 to  – 0.30; *p* = 0.18–0.65). In conclusion, our in vivo vs *post-mortem* study shows that the in vivo [^18^F]flortaucipir PET signal primarily reflects tau pathology, also at relatively low densities of tau proteinopathy, and does not bind substantially to tau neurites in neuritic plaques or in individuals with PART.

**Supplementary Information:**

The online version contains supplementary material available at 10.1007/s00401-024-02801-2.

## Introduction

The most common cause of dementia is Alzheimer’s disease (AD), accounting for 60–70% of cases [15, 34]. AD is neuropathologically characterized by the extracellular deposition of amyloid-β (Aβ) and the accumulation of intracellular insoluble paired helical filament (PHF) tau [6, 15]. Cognitive deterioration in AD is closely linked to the development of tau pathology in the brain [3] which is why it is crucial for prognostic purposes to be able to visualize in vivo tau accumulation over time. Several different tracers visualizing tau pathology in AD using positron emission tomography (PET) have been developed over the past decade [14, 21], including the only FDA-approved tau-PET tracer [^18^F]flortaucipir. In vitro autoradiography studies have shown that [^18^F]flortaucipir selectively binds to post-mortem PHFs [23, 27]. We, and others, have previously shown that in vivo [^18^F]flortaucipir signal accurately reflects post-mortem tau pathology in AD [12, 19, 24, 32, 36, 37]. However, these studies have used a rather crude measure (i.e., Braak staging) as the main measure of tau pathology, except for a few studies with small [32, 36] or modest [24] sample sizes. Braak staging, which is commonly used in neuropathological studies, allows for positivity in a region with the presence of one or a few neurofibrillary tangles, resulting in a significant variability in the amount of pathology in a positive region. Further, the Braak regions are very large, resulting in a poorer spatial resolution. A methodology utilizing a quantitative measurement and a direct region-to-region comparison should therefore result in a more reliable correlation.

There are several unaddressed questions regarding the detection level and specificity of the [^18^F]flortaucipir PET tracer. In in vivo [^18^F]flortaucipir PET studies, participants that are Aβ biomarker negative and tau-PET positive (A – T +) are occasionally reported. One possible reason for this discrepancy may be binding of [^18^F]flortaucipir to primary age-related tauopathy (PART), a disease entity with an isolated medial temporal lobe tau positivity in the absence of Aβ. Similarly, age-related and Aβ-independent correlations between neocortical tau using tau-PET tracers and cognitive measures have been reported, suggesting a possibility of PET tracers binding to PART or to tau neurites present in Aβ-plaques (neuritic plaques) even in the absence of more widespread tau pathology [4]. Further, in vivo increased [^18^F]flortaucipir uptake in temporal regions has been reported in semantic variant primary progressive aphasia (svPPA), a disease typically associated with TDP-43 proteinopathy [4, 14, 26, 35, 39, 41]. It is not yet determined if this signal represents off-target binding to TDP-43 pathology.

In this relatively large end-of-life cohort (*n* = 63), our primary objectives were to i) study the correlation between in vivo [^18^F]flortaucipir PET retention and quantitative *post-mortem* tau pathology density in corresponding brain regions; ii) determine the thresholds for detection of tau pathology with [^18^F]flortaucipir; and to iii) compare Braak staging to a quantitative measure of tau pathology. Our secondary aims were to assess whether [^18^F]flortaucipir can be used to visualize tau aggregates in Aβ neuritic plaques or in PART. Further, we wanted to evaluate if [^18^F]flortaucipir retention is correlated with concomitant TDP-43 pathology.

## Materials and methods

### Study design and participants

Data for this study were provided from the AVID A16 primary cohort study which has previously been described in detail [12]. The cohort consisted of participants with terminal illness, over the age of 50, with a projected life expectancy of less than 6 months. The cognitive function of the participants varied from normal to dementia, including diagnoses of AD and non-AD dementia (Table 1). The primary cause of death for the AVID A16 participants, as indicated by the treating physician, is specified in Supplementary Table 1. The Informant Questionnaire on Cognitive Decline in the Elderly (IQCODE) as well as Mini-Mental State Examination (MMSE) were administered with an average result of 4.5 (SD = 0.83) and 18 (SD = 11.07), respectively. The AVID A16 study was approved by institutional review boards at all study sites. The participants or authorized representatives signed informed consent to participate in the study. Four participants from the original cohort were excluded: one due to missing PET images, two because AT8-staining levels were abnormally low based on manual assessment, and one because PET and CT images were not aligned.

**Table 1:** Characteristics of the study participants

*N*	63
Age, years^α^	83.1 (9.1)
Sex, female^β^	32 (50.8%)
PET to post-mortem interval, months (± SD)	2.6 ± 2.2
Education^β^	
Middle school	1 (1.6%)
High school	26 (41.3%)
College/university	21 (33.3%)
Post-graduate school	15 (23.8%)
Clinical diagnoses^β^	
Controls	14 (22.2%)
MCI	1 (1.6%)
Dementia, AD	32 (50.8%)
Dementia, PD/DLB	7 (11.1%)
Dementia, vascular, or mixed	4 (6.3%)
Dementia, NOS*	3 (4.8%)
Dementia, FTD	2 (3.2%)
Neuropathological findings^β^	
AD	42 (66.7%)
PD/DLB	13 (20.6%)
Lewy body inclusions°	25 (39.7%)
VaD	3 (4.8%)
PSP	7 (11.1%)
HS	7 (11.1%)
CBD	2 (3.2%)
ARG	3 (4.8%)
Thal^β^	
0	4 (6.3%)
1	3 (4.8%)
2	2 (3.2%)
3	9 (14.3%)
4	7 (11.1%)
5	38 (60.3%)
Braak stage^β^	
I	2 (3.2%)
II	6 (9.5%)
III	5 (7.9%)
IV	10 (15.9%)
V	16 (25.4%)
VI	24 (38.1%)
Neuritic plaque score^β^	
0	8 (12.7%)
1	6 (9.5%)
2	2 (3.2%)
3	47 (74.6%)
ADNC^β^	
0	4 (6.3%)
1	10 (15.9%)
2	11 (17.5%)
3	38 (60.3%)

*AD* Alzheimer’s Disease: defined as dementia with Alzheimer’s disease neuropathology, at a minimum defined as intermediate or high NIA-Reagan and/or NIA-AA criteria. *PD* Parkinson’s Disease. *DLB* Dementia with Lewy bodies. *VaD* Vascular Dementia. *PSP* Progressive Supranuclear Palsy. *HS* Hippocampal Sclerosis. *CBD* Corticobasal degeneration. *ARG* Argyrophilic Grain Disease. *Dementia NOS* Dementia Not Otherwise Specified. *ADNC* = Alzheimer’s Disease Neuropathologic Change

^α^Mean (standard deviation)

^β^*N* (%)

°Lewy body inclusions in participants not diagnosed with PD or DLB

*Dementia NOS means clinical dementia was present, but not otherwise specified

### PET processing

Participants were administered ~ 370 MBq of [^18^F]flortaucipir intravenously followed by a 20-min-long PET scan (4 × 5 min acquisition frames) 80 to 100 min post-injection. A low-dose CT scan was performed for attenuation correction. Participants with cognitive decline that did not undergo autopsy within 9 months from the PET scan were either excluded from the study or underwent a repeated PET scan. Participants with normal cognition at baseline did not undergo multiple scans regardless of the time from scan to autopsy. Participants did not undergo MRI as part of the study. ROIs were manually placed on the MNI152 (Montreal Neurological Institute) template in standard space based on information from the neuropathological dissection protocol (Supplementary Fig. 1). The CT scans were spatially normalized to a CT-template in MNI152 space [20] using Advanced Normalization Tools [1]. The resulting non-linear diffeomorphic transformation was inversely applied to the ROIs, bringing them from MNI to individual CT/PET space [20]. Finally, the positions of the transformed ROIs were manually adjusted on the PET scan in each participant to account for cerebral atrophy and to correctly sample the cerebral cortex. Positioning of the ROIs on PET scans was done by a person blinded to the neuropathology results. PET standardized uptake value ratios (SUVRs) were calculated using the inferior cerebellar cortex as reference region. In line with previous work [17, 31], we used a temporal meta-ROI defined by an average of bilateral entorhinal, left superior and middle temporal gyrus, left medial temporal (BA 37), and bilateral inferolateral temporal ROIs. A previously defined cut-off for abnormality in [^18^F]flortaucipir PET in the temporal meta-ROI (1.36 [22]) was used. A larger cortical meta-ROI was composed of an average of SUVRs in: middle frontal gyrus, superior and middle temporal gyrus, inferior parietal lobule, occipital cortex (Brodmann areas (BA) 17 and 18), anterior cingulate cortex, medial temporal (BA 37), inferolateral temporal, parieto-occipital junction (BA 39), precuneus (BA 7), frontal pre-motor (BA 6), frontal anterior cortex (BA 9), orbito-frontal cortex (BA 11), and primary motor cortex (BA 4). The data were derived from the left hemisphere except for inferolateral temporal region (bilateral). A previously defined cut-off for abnormality in [^18^F]flortaucipir PET in the cortical meta-ROI was used (1.19 [22]).

### Neuropathology

Brains of study participants were collected at the respective study sites, fixed in 10% neutral buffered formalin for approximately 3 weeks, and shipped to the central study site at Banner Sun Health Research Institute, Sun City, AZ, USA, for processing. The tissue was processed according to a standardized autopsy protocol that has been specified in detail previously [2]. Specific diagnoses were made according to published criteria [10, 11, 13, 16, 25, 28, 30, 33]. A total of 19 regions were sampled in a systematic fashion and used for PET correlations. Sections were stained with hematoxylin–eosin, AT8 antibody (MN1020, ThermoFisher Scientific), and 6E10 (Covance) and the Bielschowsky silver method were used to stain for tau pathology, Aβ pathology and neuritic plaques, respectively. TAR DNA-binding protein 43 (TDP-43) pathology was assessed by pTDP-43 immunohistochemistry (Cosmobio, Ser409/410). Alzheimer’s disease neuropathological change (ADNC); Braak neurofibrillary stages, Thal phase and neuritic plaque scoring, were assessed according to National Institute on Aging—Alzheimer’s Association consensus guidelines [16, 29, 30, 38]. Low Aβ burden indicative of possible primary age-related tauopathy (PART) in study participants was defined as Thal phase = 0 (definite PART) or less than 3 (possible PART) [9], in the presence of tau pathology (Braak stages I–IV). TDP-43 immunohistochemistry was positive in at least one region in 23 participants and the presence of TDP-43 pathology was assessed semi-quantitatively (none, scarce, moderate, strong) in bilateral amygdala, entorhinal cortex/subiculum/hippocampus, and inferior temporal and orbito-frontal cortices. Each participant was assigned a TDP-43 stage according to a TDP-43 in AD staging scheme [18]. The staging algorithm was slightly modified because information from occipitotemporal cortex and middle frontal cortex was lacking, and these regions had to be omitted from the staging.

Slides were scanned using a Hamamatsu NanoZoomer digital scanner (Hamamatsu Photonics, Japan), at 80 times magnification. For AT8 immunohistochemistry (IHC), every region of interest was sampled at 1–3 locations at 5–10 times magnification (the number of images and magnification was kept constant for all participants within each region). To estimate AT8 pathology, all images were digitally processed by applying a red–green filter to reduce the red haematoxylin counterstain. The true binding was then assessed by filtering out background noise by applying a threshold to the neuropathology images. Due to variations in staining intensity between study participants, an individual threshold was set manually for each participant. The thresholds were set by visually inspecting the staining intensity and finding the threshold where tau neurites and neurofibrillary tangles remained, but a general diffuse background disappeared. Thresholding was performed by a person blinded to the PET results and clinical status of the participant. Subsequently, the percentage of AT8-positive pixels for each image was calculated. The manual background reduction method was validated through an alternative method where pathology was segmented from non-pathology in an automated fashion (Supplementary Fig. 2 and 3). Each image was then pre-processed by first converting pixels with a high red intensity (pixel value > 100) to white and then converting the whole image to grayscale. For the segmentation, a graph cut algorithm was used, a computational technique that can be seen as a practical implementation of Markov Random Fields (MRF) principles [5].

### Statistical analysis

All analyses were performed in RStudio (Version 2023.09.1 + 494) and R (version 4.3.2). *P* < 0.05 (two-sided) was considered statistically significant. Mean and standard deviations were used to describe the demographic characteristics of the study participants. The correlations between [^18^F]flortaucipir SUVR values and neuropathology were assessed using Spearman ⍴ as the data were not always normally distributed. Spearman ⍴ was also used for assessing the correlation between ordinal variables (Thal phase, neuritic plaque scores, and TDP-43 stages) and [^18^F]flortaucipir SUVR values. To assess linearity between PET values versus Braak stages and pathology divided into sextiles, linear models and polynomial regression models were compared with regards to residual sum of squares and Akaike information criterion using *R*. Statistical significance was assessed with F test for Nested models using the “pf “ function in *R*. Youden index was used to establish detection thresholds for abnormality on neuropathological assessment depending on the thresholds for [^18^F]flortaucipir PET in the temporal meta-ROI and cortical meta-ROI, respectively.

## Results

### Participants

A total of 63 participants were included. Demographic information is presented in Table 1. The mean age was 83.1 (± 9.1) years, 50.8% were female, 66.7% of participants had AD, defined as having both a clinical diagnosis of dementia and AD neuropathology. Furthermore, 60.3% of participants had widespread Aβ pathology (Thal phase of 5), 63.5% had a tau spread beyond the temporal lobes (Braak stage > IV), 74.6% had a neuritic plaque score of 3, and 60.3% had Alzheimer’s disease neuropathologic change (ADNC) of 3.

### Correlations and concordance between [^18^F]flortaucipir PET and quantitative neuropathology when measured in the same regions

**Fig. 1:**
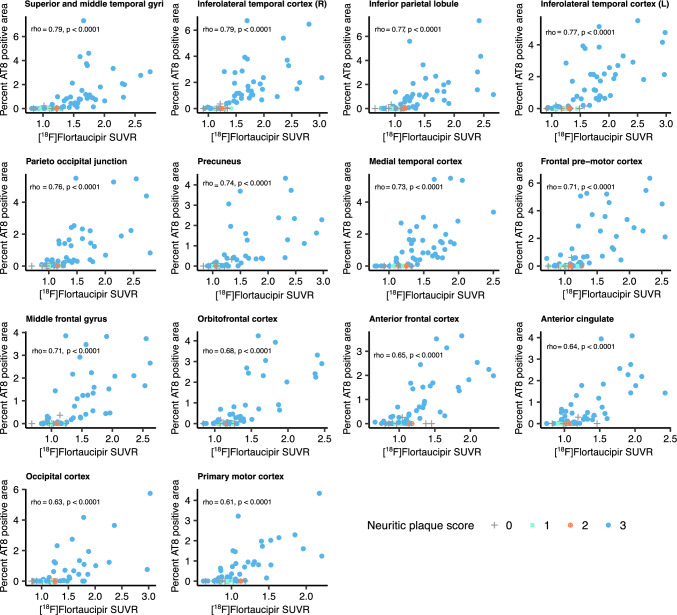
Correlations between %AT8-positive area in neuropathology images and [^18^F]flortaucipir SUVR. Correlations between PET SUVR and % AT8-positive area in neuropathology images in corresponding cortical brain regions. *SUVR* Standardized Uptake Value Ratio. Neuritic plaque score according to NIA-AA [16]. Grey cross = 0, cyan square = 1, orange cross-circle = 2, blue circle = 3

**Fig. 2:**
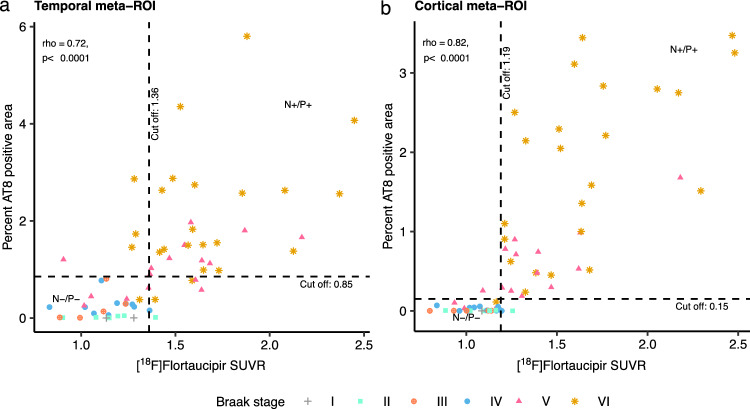
Correlation between %AT8-positive area in neuropathology images and [^18^F]flortaucipir SUVR in composite cortical regions. Correlation between PET and pathology in a temporal meta-ROI (**a**) and a more extensive cortical meta-ROI (**b**) with additional visualization of Braak stages. The dashed vertical line indicates the cut-off for flortaucipir SUVR positivity (1.36 in the temporal meta-ROI; 1.19 in cortical meta-ROI). The dashed horizontal line represents the cut-off for AT8-positive area at 0.85% for the temporal meta-ROI (**a**) and 0.15% for the larger cortical ROI (**b**). N – /P – : tau-negativity both in neuropathology and PET. N + /P + : tau positivity both in neuropathology and PET. Braak stages: grey cross = I, cyan square = II, orange cross-circle = III, blue circle = IV, red triangle = V, golden star = VI. The temporal meta-ROI is defined by an average of entorhinal (bilateral), superior and medial temporal gyrus (left), medial temporal (BA 37; left), and inferolateral temporal (bilateral) ROIs. The cortical meta-ROI is defined by an average of left sided (unless indicated as bilateral) ROIs: middle frontal gyrus, superior and middle temporal gyrus, inferior parietal lobule, occipital cortex (BA 17 and 18), anterior cingulate cortex, medial temporal (BA 37), inferolateral temporal (bilateral), parieto-occipital junction (BA 39), precuneus (BA 7), frontal pre-motor (BA 6), frontal anterior cortex (BA 9), orbito-frontal cortex (BA 11), and primary motor cortex (BA 4). *ROI* Region of Interest

**Fig. 3:**
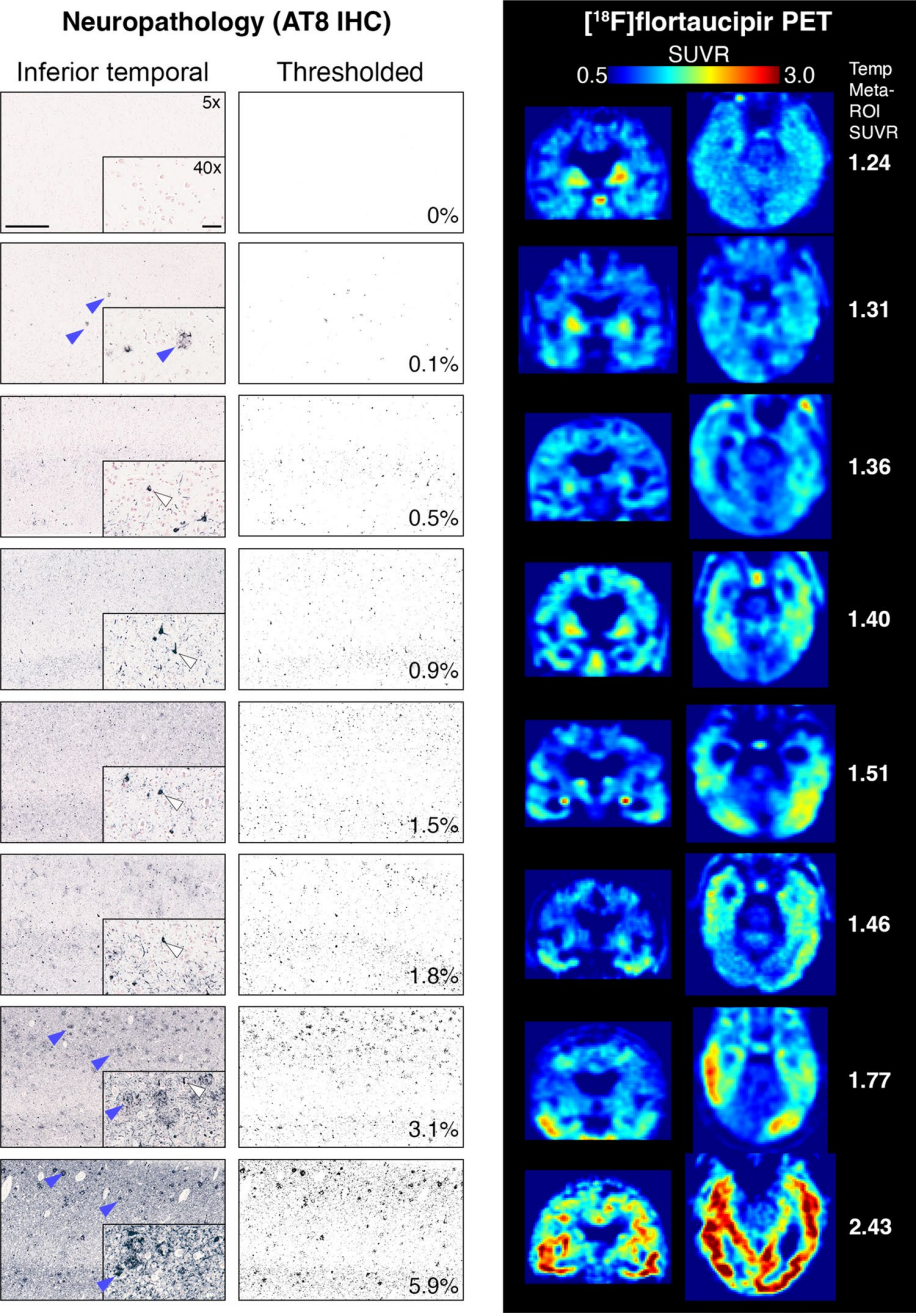
Visualization of different levels of tau pathology and corresponding [^18^F]flortaucipir PET images. The leftmost panel shows AT8 IHC in the inferolateral temporal cortex at 5 × or 40x (insets) magnification. The “Thresholded” panel shows the images resulting after thresholding and the values indicate the % positive area in the image. The right-hand panel shows corresponding transversal and coronal PET SUVR images through the temporal lobes. In the rightmost column, temporal meta-ROI SUVRs are indicated for the corresponding PET images. *IHC* immunohistochemistry. Scale bars represent 500 µm (5x) or 50 µm (40x). Blue arrowheads indicate examples of tau in neuritic plaques and white arrowheads examples of neurofibrillary tangles. *ROI* Region of Interest. *SUVR* Standardized Uptake Value Ratio

[^18^F]Flortaucipir PET SUVRs in the neocortex were compared to neuropathological sections in matching regions. The PET signal showed moderate-to-strong correlations to AT8 neuropathology in all studied neocortical regions (spearman rho = 0.61–0.79, *p* < 0.0001; Fig. 1). In the temporal meta-ROI the correlation between [^18^F]flortaucipir and AT8-positive area was moderate-to-substantial (rho = 0.72, *p* < 0.0001, Fig. 2, Supplementary Fig. 4). Using a previously defined cut-off (1.36 [22]) for abnormality of [^18^F]flortaucipir PET in the temporal meta-ROI, we established a detection threshold of 0.85% for AT8 pathology corresponding to when abnormal PET-levels were detected. To give an indication of how much pathology this represents, we have included images of AT8 pathology at low and high resolution along with the thresholded images from the same region and transversal and coronal sections of the corresponding [^18^F]flortaucipir PET images (Fig. 3). All individuals above the threshold for [^18^F]flortaucipir and AT8 abnormality (Neuropathology + /PET +) had a neuritic plaque score of 3 (Supplementary Fig. 4a), as well as a Braak score of V to VI (Fig. 2a). Still, there was a substantial variability in the amounts of pathology in the Braak stage V and VI groups (y-axes, Fig. 2). The concordance between PET and neuropathology positivity was high, with an accuracy of 0.841 (0.727–0.921, 95% CI). Similar results were observed in a larger cortical meta-ROI encompassing large parts of the neocortex (rho = 0.82, *p* < 0.0001, Fig. 2b). Using a cut-off for abnormality in [^18^F]flortaucipir PET SUVR of 1.19 [22], resulted in a cut-off for detection of AT8 abnormality at 0.15% in this cortical meta-ROI. All individuals above the threshold for neuropathology and PET (Neuropathology + /PET +) had a neuritic plaque score of 3 (Supplementary Fig. 4b) as well as a Braak stage of V or VI (Fig. 2b). The concordance between PET and neuropathology was high with an accuracy of 0.937 (0.845–0.982, 95% CI). A generalized linear model using [^18^F]flortaucipir in the temporal and cortical meta-ROIs as the outcome and tau neuropathology, sex, age, and PET to post-mortem interval as predictors indicated that tau neuropathology was the strongest predictor, with additional contributions from sex and age (Table 2).

**Table 2:** Results from generalized linear models

	Estimate	*T* value	*P* value
Temporal meta-ROI			
Tau neuropathology	0.227	7.42	5.7 × 10^–10^
Age	– 0.009	– 2.87	0.006
Sex (male)	– 0.184	– 3.12	0.003
PET-post-mortem interval	– 0.0004	– 1.10	0.28
Cortical meta-ROI			
Tau neuropathology	0.296	9.68	1.0 × 10^–13^
Age	– 0.006	– 2.33	0.023
Sex (male)	– 0.139	– 2.76	0.008
PET-post-mortem interval	– 0.0005	– 1.43	0.16

A generalized linear model with [^18^F]flortaucipir PET SUVR ~ Tau neuropathology + age + sex + PET-post-mortem interval was analyzed. To account for the data not being normally distributed, a model with a gamma distribution was used. The temporal meta-ROI is defined by an average of bilateral entorhinal, left superior and medial temporal gyrus, left medial temporal (BA 37), and bilateral inferolateral temporal ROIs. The cortical meta-ROI is defined by an average of left sided (unless indicated as bilateral) ROIs: middle frontal gyrus, superior and middle temporal gyrus, inferior parietal lobule, occipital cortex (BA 17 and 18), anterior cingulate cortex, medial temporal (BA 37), inferolateral temporal (bilateral), parieto-occipital junction (BA 39), precuneus (BA 7), frontal pre-motor (BA 6), frontal anterior cortex (BA 9), orbito-frontal cortex (BA 11), and primary motor cortex (BA 4)

*ROI* Region of Interest. *SUVR* Standardized Uptake Value Ratio

### Comparison of [^18^F]flortaucipir PET, quantitative neuropathology, and Braak staging

**Fig. 4:**
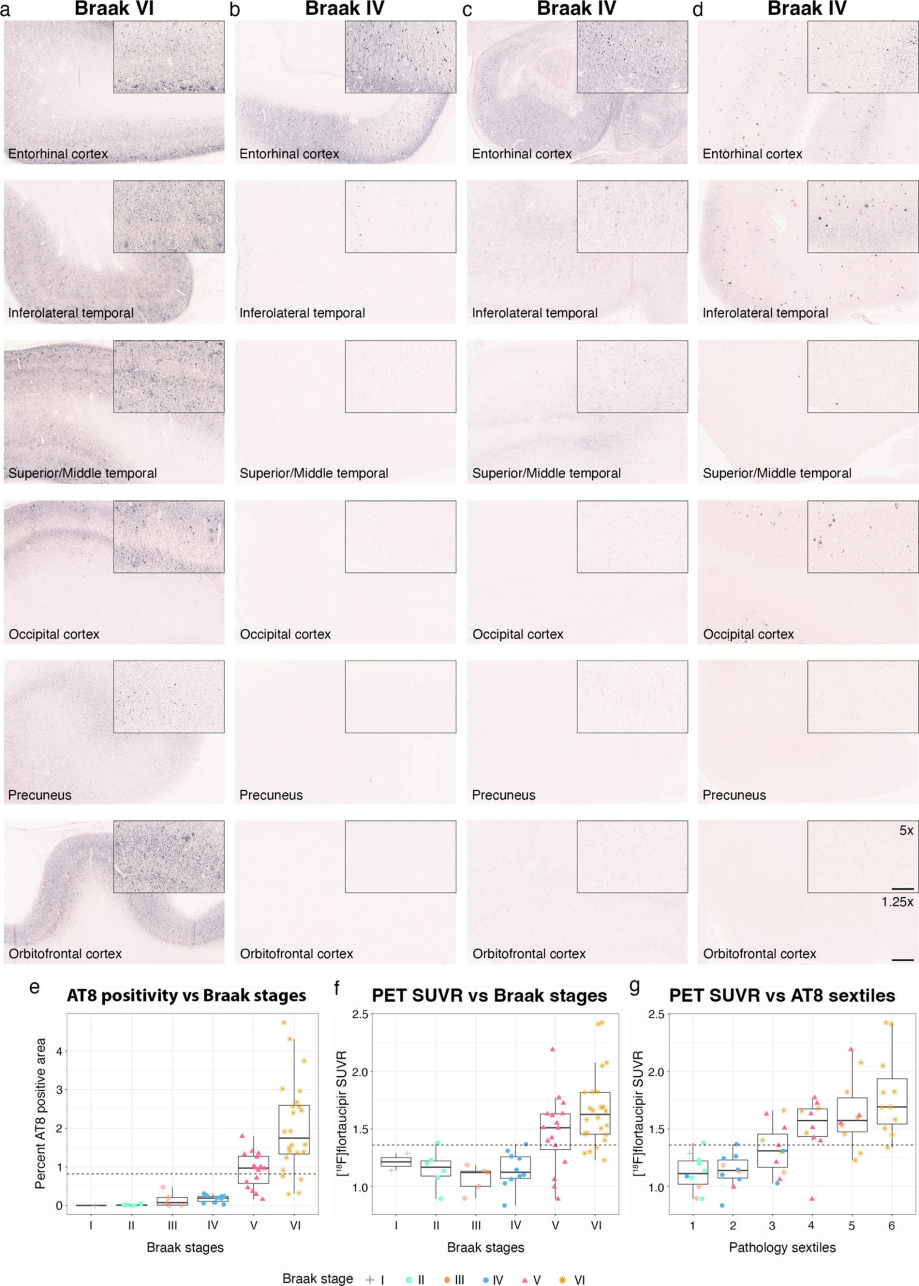
Tau pathology in different Braak stages. **a**–**d** Neuropathology AT8 immunohistochemistry images from one individual with Braak stage VI (panel **a**) and three individuals with Braak stage IV (panels **b**–**d**). Images are shown at 1.25 × magnification (scalebar 1 mm) with a 5 × magnification inset (scale bar 500 µm). The plot in panel **e** shows the tau pathology load in the temporal lobe depending on Braak stages. The graph in panel **f** shows the [^18^F]flortaucipir PET retention in the temporal meta-ROI plotted in the different Braak stages and panel **g** shows the [^18^F]flortaucipir PET retention in temporal meta-ROI against quantitative AT8 neuropathology divided into sextiles based on the tau pathology load. The horizontal dashed line in **e** represents the quantitative cut-off for tau pathology (0.85%). The horizontal dashed line in **f**, **g** represents the quantitative cut-off for [^18^F]flortaucipir PET in temporal meta-ROI (1.36). *SUVR* Standardized value uptake ratio. Braak stages: grey cross = I, cyan square = II, orange cross-circle = III, blue circle = IV, red triangle = V, golden star = VI

It has previously been reported that [^18^F]flortaucipir PET does not detect tau pathology earlier than Braak stage V, which has caused concerns for the sensitivity of the tracer to detect early tau pathology. The Braak-staging system, however, allows for positivity with the detection of single or few neurofibrillary tangles in a region, suggesting that the system is not very quantitative and not linearly related to the overall tau load in the brain. Figure 4 shows representative images from one Braak VI individual (Fig. 4a) and three Braak IV individuals (Fig. 4b-d). These panels indicate that there is substantial variation in the amount of pathology among the Braak IV cases, and the amount of tau tangles in individuals with Braak stage IV was sparse even in inferolateral temporal cortex and medial temporal gyrus compared to the individual with Braak stage VI. When plotting the quantitative AT8 values according to Braak stages (Fig. 4e), we find only minimal amounts of tau pathology in the temporal lobe in Braak stages < V. We next compared the PET signal against the Braak-staging system and against quantitative AT8 pathology divided into sextiles based on the amount of tau pathology detected. As indicated by the plot in Fig. 4f, the Braak stages do not represent a linear function of pathology (a polynomial regression described the data significantly better than a linear model, *p* = 0.0067, ΔAIC – 5.8; Supplementary Fig. 5). A quantitative AT8 evaluation of pathology, where participants were divided into sextiles based on levels of neuropathology (Fig. 4g), showed a visually better linear fit and data were not better described by a polynomial model (*p* = 0.54; ΔAIC + 1.6; Supplementary Fig. 5).

### Correlations to low levels of tau and relation of tau to [^18^F]flortaucipir off-target binding

**Fig. 5:**
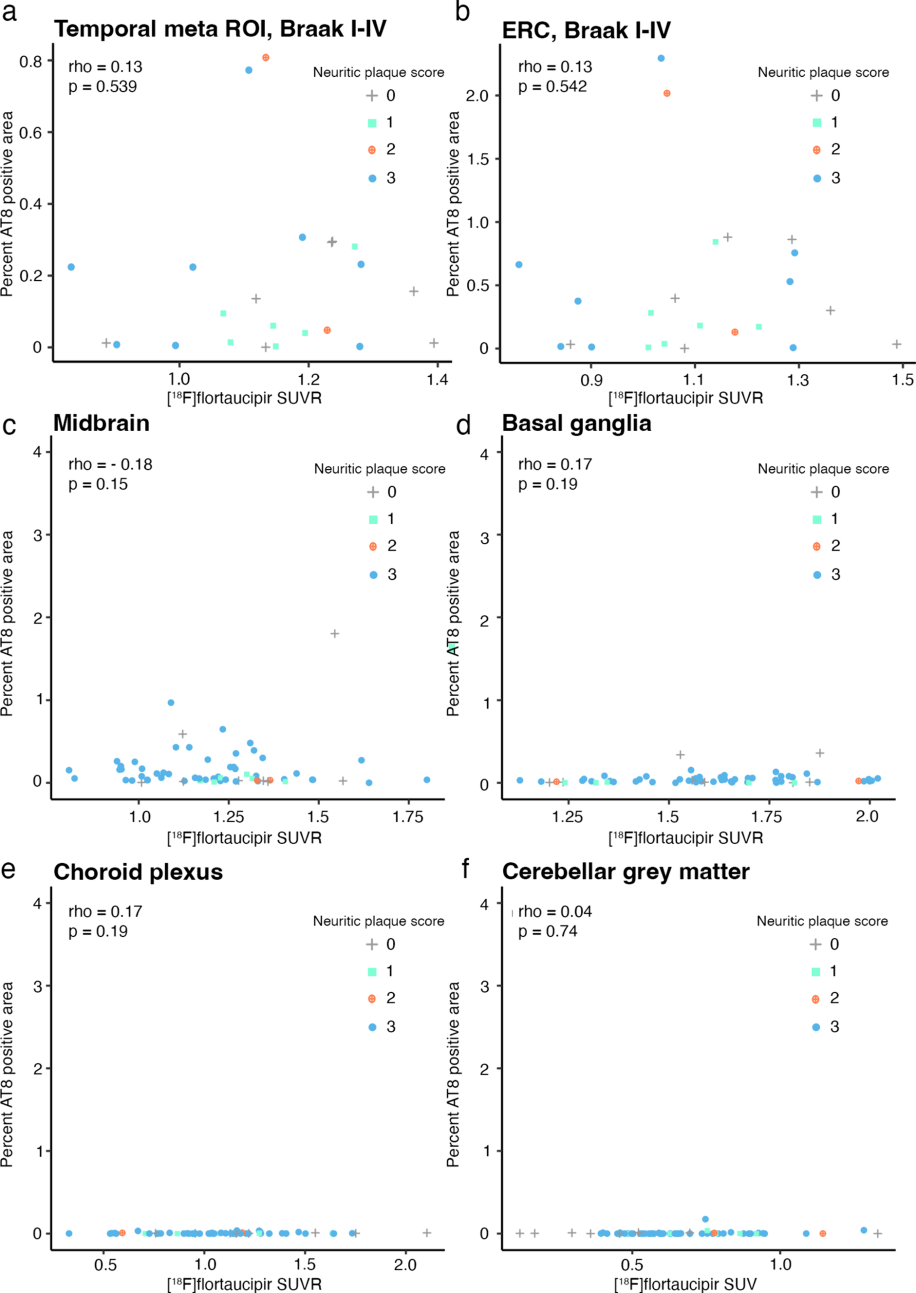
[^18^F]Flortaucipir uptake in patients with early AD pathology and in off-target regions. Correlations between %AT8-positive area and [^18^F]flortaucipir SUVR in **a** a temporal meta-ROI and **b** bilateral entorhinal cortex in individuals with Braak stages I–IV. Panels **c**–**e** show the correlation between %AT8-positive area and [^18^F]flortaucipir SUVR in off-target regions: **c** Midbrain, **d** the Basal Ganglia, and **e** Choroid plexus. Plot **f** shows [^18^F]flortaucipir SUV and % AT8-positive area in the cerebellar grey matter, a widely used as reference region. Neuritic plaque score according to NIA-AA [16], grey cross = 0, cyan square = 1, orange cross-circle = 2, and blue circle = 3

To evaluate whether there could be sub-threshold positive correlations between [^18^F]flortaucipir retention and early tau pathology in the ERC, we plotted the PET and %AT8 pathology data from the ERC and the temporal meta-ROI separately in participants with Braak stages I–IV (Fig. 5a, b). In this analysis, we did not find any significant correlation between AT8 tau and [^18^F]flortaucipir (temporal meta-ROI, rho = 0.13, *p* = 0.54; ERC, rho = 0.11, *p* = 0.60). Neither did we find any correlations between tau pathology and PET in the ERC or amygdala in patients with neuropathological tau (Braak stages I–IV) but low Aβ levels (as indicated by Thal phase < 3), indicative of possible primary age-related tauopathy (PART; Supplementary Fig. 6; Supplementary Table 2).

Next, we aimed to study whether [^18^F]flortaucipir to some degree might reflect tau-positive neurites in plaques. To avoid the influence of neurofibrillary tau we assessed the correlations of neuritic plaque burden in extratemporal regions in participants with tau limited to Braak stages I–IV, that is, with neurofibrillary tau restricted to the temporal lobes. We did not observe significant correlations between [^18^F]flortaucipir retention and Aβ neuritic plaque scores in regions outside of the temporal cortex in participants with Braak stage I–IV (rho-values =  – 0.16 – 0.12, *p* = 0.48–0.61), indicating that [^18^F]flortaucipir is not sensitive enough to pick up potential tau neurites present in neuritic plaques (Supplementary Fig. 7). It is well established that [^18^F]flortaucipir PET has an in vivo off-target binding in the substantia nigra, the basal ganglia and the choroid plexus. When assessing AT8-positive area in these regions, we found no correlation between the PET signal detected in these off-target regions and underlying tau pathology (Midbrain, rho =  – 0.18, *p* = 0.15; Basal ganglia, rho = 0.17, *p* = 0.19; Choroid plexus, rho = 0.17, *p* = 0.19; Fig. 5c–e), suggesting that the off-target [^18^F]flortaucipir signal comes from other sources than tau pathology. Overall, the levels of tau detected in the off-target regions were limited. Similarly, no significant tau pathology was detected in the cerebellar grey matter (Fig. 5f).

Additionally, since [^18^F]flortaucipir retention has been reported in svPPA [14, 35, 39, 42], linked to TDP-43 pathology rather than tau, we analyzed the correlation between [^18^F]flortaucipir SUVRs and TDP-43 in participants that were TDP-43 positive in any region at autopsy. We observed no statistically significant correlations in the amygdala, ERC, or using a composite region consisting of amygdala, ERC, orbito-frontal cortex and the inferolateral temporal lobe (Supplementary Fig. 8; Supplementary Table 3).

## Discussion

In this study, we demonstrate moderate-to-strong linear correlations between [^18^F]flortaucipir SUVRs and tau neuropathology assessed by AT8-positive areas in corresponding neocortical ROIs. We found that that the detection limit for [^18^F]flortaucipir was 0.85% AT8-labelled pathology in a temporal meta-ROI and 0.15% in a larger cortical meta-ROI. Tau pathology in Braak stages < V was not reliably detected using the meta-ROI, most likely due to levels of pathology being below the detection threshold in these early stages. We further show that [^18^F]flortaucipir does not detect tau pathology associated with Aβ-related neuritic plaques and cannot reliably distinguish tau pathology associated with PART from background signal. In addition, we observe that the retention seen in the substantia nigra, the choroid plexus, and the basal ganglia is not related to tau pathology and that co-occurring TDP-43 pathology does not seem to influence the [^18^F]flortaucipir signal. The present study indicates that [^18^F]flortaucipir can detect tau proteinopathy in a linear fashion which is important when using the method for either estimating the total load of tau proteinopathy in the brain, or when determining the amount of tau removed by an intervention in clinical trials.

[^18^F]Flortaucipir PET has been linked to positive visual read assessments in individuals with neuropathological Braak stages V–VI [12], but it has remained unclear whether the method is sensitive enough to detect tau at lower levels. We found a moderate-to-strong correlation in a temporal meta-ROI overall, but we did not observe a correlation when evaluating participants at Braak stages I–IV separately. This was likely because these Braak I–IV individuals exhibit a low density of tau proteinopathy in the temporal lobes, with AT8-staining in less than 0.85% of the area within the temporal composite region. Consequently, our study suggests that [^18^F]flortaucipir does not detect tau at Braak stages < V due to sub-threshold levels of tau at these early disease stages. The findings are in line with a previous study showing elevated [^18^F]flortaucipir PET SUVR in study participants with Braak stages of V or greater [24]. To understand the Braak-staging system and whether the categories represent a meaningful quantitation of the pathology, we plotted [^18^F]flortaucipir PET retention according to Braak staging as well as according to level of pathology divided into sextiles, determined by the quantitative %AT8 IHC method. Visually and statistically, the Braak-staging system had a non-linear relation to the PET retention, whereas grouping according to the density of tau proteinopathy sextiles resulted in a clearer linear relationship, indicating that [^18^F]flortaucipir indeed is a better measure of the local load of tau proteinopathy than Braak staging.

In general, we observed moderate-to-strong correlations between [^18^F]flortaucipir PET and cortical %AT8 neuropathology in the different neocortical regions studied, which is congruent with previous smaller studies [24, 32, 36]. Based on quantitative %AT8 pathology, we could determine a detection threshold of pathology at ~ 0.85% in a temporal meta-ROI and ~ 0.15% in a cortical meta-ROI, corresponding to scattered, but clearly identifiable neurofibrillary tangles and dystrophic neurites (see Fig. 3). To rule out that the correlation was not only due to a difference between [^18^F]flortaucipir PET negative and positive individuals, we performed a sensitivity analysis of PET to neuropathology correlations in participants positive for tau neuropathology, positive in the [^18^F]flortaucipir PET or both. We found similar correlations in these subgroups (Supplementary Fig. 9). Due to the single cohort nature of the current study, the exact detection thresholds will need to be validated in an independent cohort.

There are several reports of tau positivity in participants that are Aβ-negative (A – T +) in the literature [8, 40]. This can of course be due to cut-offs for Aβ-positivity being too high or cut-offs for tau positivity being too low, but at least in the medial temporal regions, it could potentially also represent binding to tau proteinopathy in the case of PART. To determine whether [^18^F]flortaucipir can reliably detect tau proteinopathy in cases with possible PART we assessed [^18^F]flortaucipir uptake in participants with low Aβ and evidence of neuropathological tau deposits. As in previous studies, definitive PART was defined as Thal phase = 0, and possible PART as Thal phase 1–2, in combination with Braak NFT stage I–IV [9]. Similar to previously published reports [19, 24], we did not observe any correlations between [^18^F]flortaucipir PET and tau neuropathology in cases with low Aβ burden (Supplementary Fig. 6), indicating that PART cannot reliably be separated from the background signal using this PET method. Many of the PART participants showed very low quantitative levels of tau, which could in part explain the difficulties in detecting any [^18^F]flortaucipir signal in these cases. Additionally, to compare [^18^F]flortaucipir SUVR with visual read, we determined the threshold for detection of tau pathology using visual read to be 0.27% in the temporal meta-ROI and 0.11% in the cortical meta-ROI compared with 0.85% and 0.15% using [^18^F]flortaucipir SUVR, indicating that visual read could be more sensitive than [^18^F]flortaucipir SUVR (Supplementary Table 4).

We further evaluated whether [^18^F]flortaucipir could potentially detect early tau-positive neurites present in Aβ plaques before neurofibrillary tangles are formed. To do this, we assessed the correlation between [^18^F]flortaucipir PET and regional neuritic plaque score in neocortical regions outside the temporal lobe in participants with tau neurofibrillary tangles restricted to the temporal lobe (Braak stages I–IV). We found no significant correlation between plaque density scores and [^18^F]flortaucipir retention in any of the assessed extratemporal regions (Supplementary Fig. 7).

A secondary aim was to assess the correlation between pathology and [^18^F]flortaucipir in off-target binding regions. We demonstrated a lack of correlation between [^18^F]flortaucipir binding and tau pathology in several off-target ROIs such as the midbrain, the basal ganglia, and the choroid plexus. This is in line with a previous study that reported no significant correlation between [^18^F]flortaucipir SUVR and tau neuropathology in the basal ganglia [32]. We further report minimal tau pathology in the cerebellar grey matter, only one case showed low-level pathology in this region. Cerebellum is widely used as a PET reference region as specific uptake of [^18^F]flortaucipir has been assumed to be negligible. In support of this notion, we find very limited neuropathological tau pathology in the cerebellar cortex in this material (Fig. 5f).

Additionally, we conducted an analysis of TDP-43 pathology as previous studies showed increased temporal [^18^F]flortaucipir uptake in svPPA [4, 14, 26, 35, 39, 41], a disease typically caused by TDP-43 proteinopathy. We have previously shown low-level temporal [^18^F]flortaucipir retention in patients with svPPA but very limited retention in *C9orf72* mutation carriers, which induces TDP-43 proteinopathy [35]. Using autoradiography, we previously found no specific binding of ^3^H-flortaucipir to TDP-43 pathology neither in svPPA, nor in subjects with *C9orf72* mutations. In the current dataset, we found no correlation between [^18^F]flortaucipir retention and TDP-43 pathology burden when present as a co-occurring pathology with AD, indicating no specific binding of [^18^F]flortaucipir to TDP-43. However, it should be noted that there were no svPPA cases included in the study and that the increased retention seen in the correlation plots is derived from Aβ-positive/tau-positive individuals. These findings are in line with a recently published article showing a relationship between medial temporal volume loss and TDP-43 pathology, but no association of TDP-43 pathology with [^18^F]flortaucipir retention [7]. Other reasons for in vivo [^18^F]flortaucipir retention in svPPA could for example be off-target binding to monoamine oxidase-B or neuroinflammatory processes. These possibilities were not further investigated in this work.

There are both strengths and limitations to this study. The main strengths are the number of brain regions studied, the use of detailed quantitative measurements of post-mortem tau pathology, and the fact that the neuropathology sections and in vivo PET scans are from corresponding brain regions, making this the most comprehensive study of [^18^F]flortaucipir and quantitative neuropathology so far.

There are, however, several limitations. First, the PET ROIs were placed on PET scans according to the standardized neuropathological dissection manual and not guided by the exact location of neuropathology sampling for each individual participant. Further, due to the multisite nature of data collection, different PET scanners with different resolution and reconstruction protocols were used. This likely introduced some noise into the correlation data due to mismatching of PET and pathology ROIs that we cannot fully compensate for. The results in small regions, such as the entorhinal cortex, also become less robust and results should be interpreted with this in mind. A representative example of the ERC ROI has been included as Supplementary Fig. 10. Second, the neuropathology images were adjusted for background IHC intensity through a manual thresholding process, which could possibly introduce bias. Therefore, as a second method of filtering out background noise in neuropathology images, we used Markov random field segmentation (Supplementary Figs. 2, 3). Markov random fields’ segmentation reduces the AT8-positive area and filters out more signal, but overall, the results remain highly comparable between the two methods. Third, the sample sizes were small when it comes to the subpopulations with TDP-43 pathology (*n* = 22), PART (*n* = 8), or Braak I–V stages (*n* = 23). Due to lower power, correlations in these subgroups may have been underestimated and should therefore be considered preliminary until replicated in a larger sample size. Fourth, analysis of TDP-43 was not suited for automated quantitative analysis due to low overall levels of pathology; therefore, a cruder semiquantitative grading of the pathology was used. Fifth, even though we found no correlations between [^18^F]flortaucipir and PART-related tau, we cannot rule out that potentially visual reads of the medial temporal lobes could represent a more sensitive mode for detecting PART pathology. Finally, three of the cases in this study have been published in a detailed correlation analysis previously [32]. However, in relation to the previously published data, the quantitative data generated in this study and the methods used are novel.

In conclusion, we find that [^18^F]flortaucipir correlates well with cortical tau proteinopathy and that this correlation is linear with quantitative measures of pathology. The detection limit of [^18^F]flortaucipir corresponds to neuropil thread tau and neurofibrillary tangles present in ~ 0.85% of the surface area in a temporal meta-ROI and ~ 0.15% in a cortical meta-ROI, which corresponds to scattered but clearly visible tau pathology in neuropathological sections. Further, we found no indications that [^18^F]flortaucipir binds to Aβ neuritic plaques or TDP-43 inclusions and we demonstrate that the off-target binding in the substantia nigra, basal ganglia, and choroid plexus is unrelated to tau proteinopathy and that the cerebellum constitutes a suitable reference region for in vivo PET imaging.

## Supplementary Information

Below is the link to the electronic supplementary material.

Supplementary file1 (DOCX 3619 KB)
