# Supplementary material for: Association of in vivo retention of [18f] flortaucipir pet with tau neuropathology in corresponding brain regions
Source: Acta Neuropathol. 2024 Sep 19;148(1):44. doi: 10.1007/s00401-024-02801-2 (PMC11413084; doi:10.1007/s00401-024-02801-2)
Supplement: Supplementary file 1 — Supplementary file1 (DOCX 3619 KB) [file 401_2024_2801_MOESM1_ESM.docx]

Supplementary information

ASSOCIATION OF IN VIVO RETENTION OF [^18^F]FLORTAUCIPIR PET WITH THE DENSITY OF TAU NEUROPATHOLOGY IN CORRESPONDING BRAIN REGIONS

Tove Freiburghaus, MD^1*^, Daria Pawlik, MD^1,2*^, Kevin Oliveira Hauer, MD^1^, Rik Ossenkoppele, PhD^1,4,5^, Olof Strandberg, PhD^1^, Antoine Leuzy, PhD^1^, Jonathan Rittmo, MSc^1^, Cécilia Tremblay, PhD^3^, Geidy E. Serrano, PhD^3^, Michael J. Pontecorvo, PhD^6^, Thomas G. Beach, MD, PhD^3^, Ruben Smith, MD, PhD^1,7**§^, Oskar Hansson MD, PhD^1,7**§^

**Index Page**

**Supplementary Table 1** - Primary causes of death in AVID A19 study 2

**Supplementary Figure 1** – ROIs superimposed on a MNI152 MRI template 3-4

**Supplementary Figure 2** – Results with neuropathology estimated by Markov Random Fields in individual cortical regions of interest 5

**Supplementary Figure 3** – Results with neuropathology estimated by Markov Random Fields in temporal and cortical meta-regions of interest 6-7

**Supplementary Figure 4** – Temporal and cortical meta-ROI with neuritic plaque score annotation 8

**Supplementary Figure 5** – Residuals and comparisons of linearity of [^18^F]flortaucipir PET vs Braak stages/Pathology sextiles 9

**Supplementary Figure 6** – ERC and amygdala SUVRs in participants with low amyloid-beta 10

**Supplementary Table 2** - Characteristics of the participants with possible or probable PART 11

**Supplementary Figure 7** – Correlations between neuritic plaque scores and [^18^F]flortaucipir SUVR in extratemporal regions in participants with Braak stages ≤ IV. 12

**Supplementary Figure 8** – Correlation of TDP-43-stage and [^18^F]flortaucipir SUVRs 13

**Supplementary Table 3** - Characteristics of the study participants positive for TDP-43 14

**Supplementary Figure 9** - – PET to neuropathology correlations within pathology and PET positive subjects

15

**Supplementary Table 4** - Thresholds for detection of tau pathology using [^18^F]flortaucipir SUVR and visual read 16

**Supplementary Figure 10** – Representative example of the ERC ROI localized on an individual [^18^F]flortaucipir PET image. 17

**Refererences** 18

**Supplementary Information**

**Supplementary Table 1**

Primary causes of death in AVID A16 study

|  | Controls (n = 14) | MCI (n=1) | Dementia (n=48) |
| --- | --- | --- | --- |
| End stage dementia disorder |  |  | 33 |
| Cancer | 7 | 1 | 3 |
| Cardiac disease ^a^ | 5 |  | 3 |
| Lung disease ^b^ | 1 |  | 3 |
| Renal Failure |  |  | 2 |
| Pneumonia | 1 |  | 1 |
| Motorneuron disease |  |  | 1 |
| Intracerebral hemorrhage |  |  | 1 |
| Not specified |  |  | 1 |

^a^ Including congestive heart failure, “cardiovascular accident” and “complete heart block”.  ^b^ Including chronic obstructive pulmonary disease, interstitial lung disease and respiratory failure.

**Supplementary Figure 1**

**
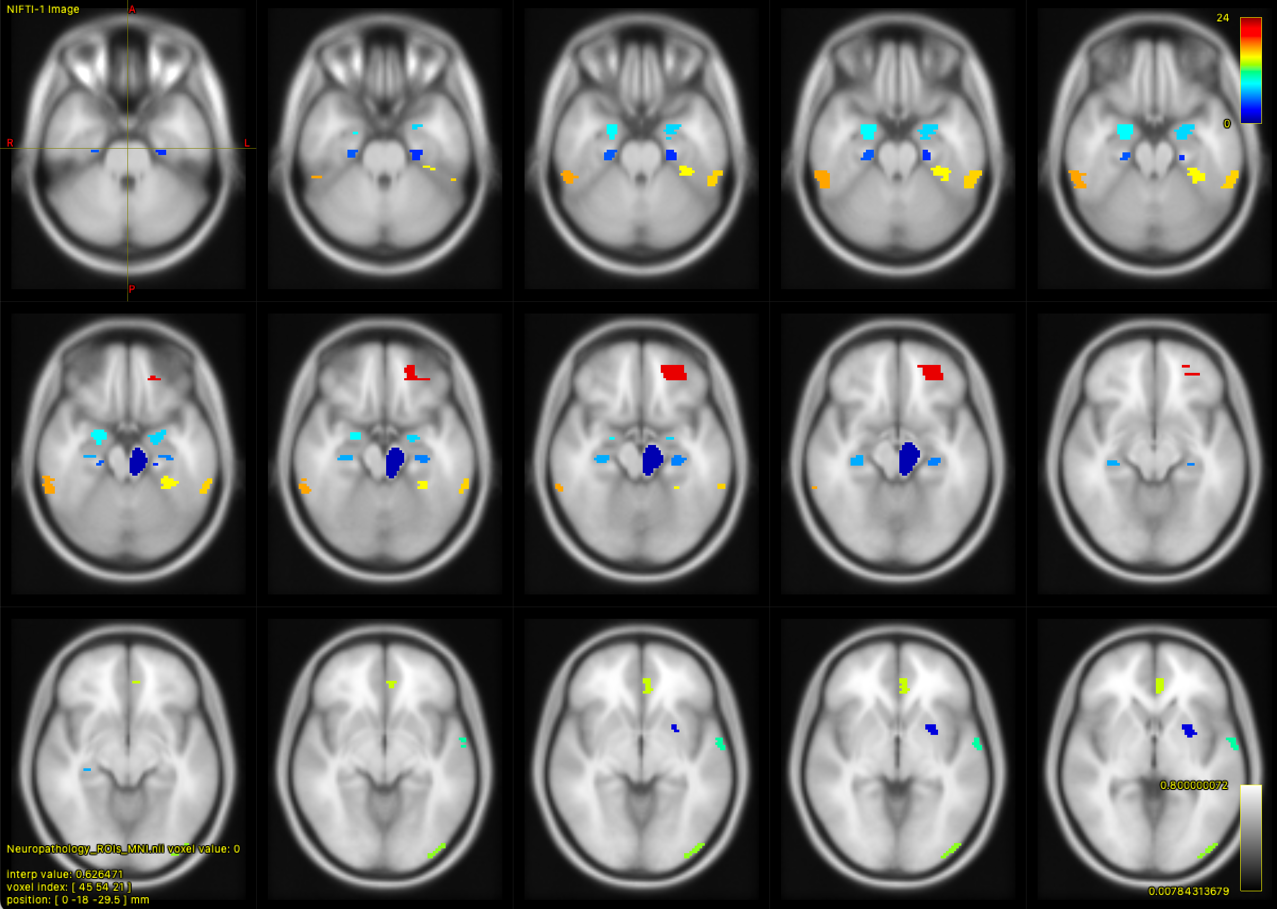
**

**
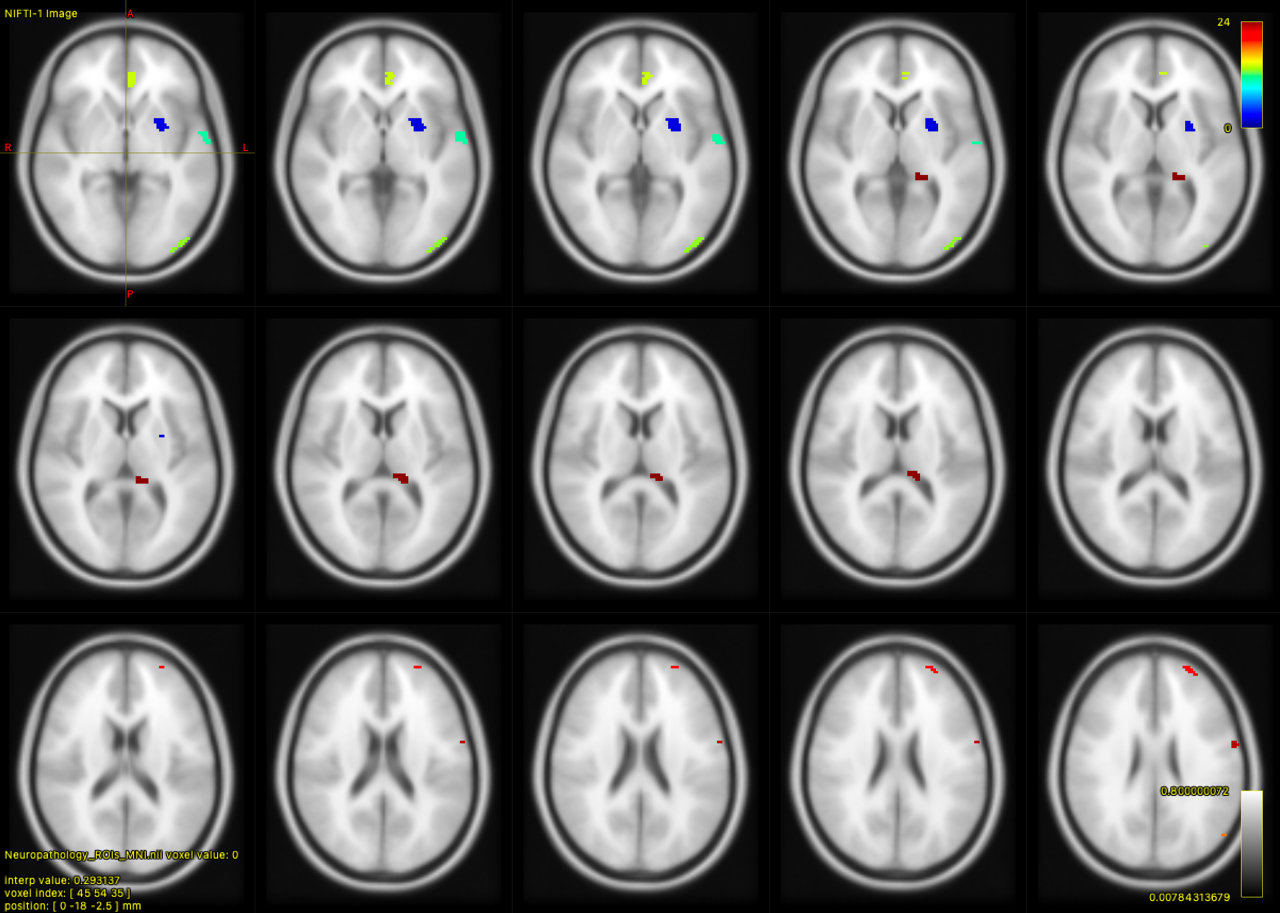
**

**
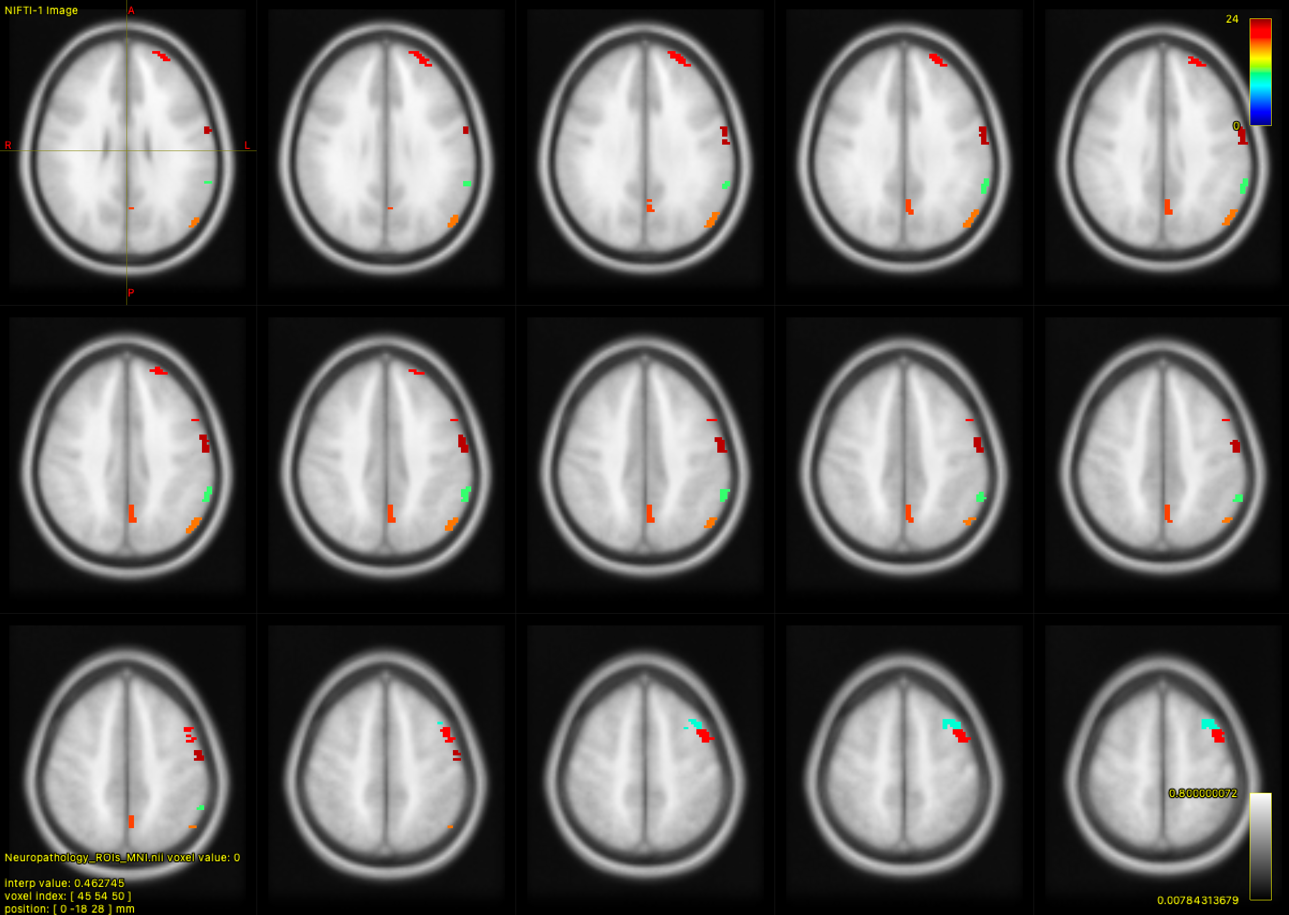
**

Transversal images of the analyzed ROIs overlaid on an MNI152 MR template. Please note that the PET ROIs were intentionally small to as closely as possible match the areas of neuropathological sampling. All ROIs were manually adjusted after warping out into individual patient space to sample cortical areas of the PET image. The manual adjustment was made by a person blinded to the neuropathological results. All regions were sampled on the left side unless specified as bilateral. Midbrain (dark blue), basal ganglia (putamen; dark blue), entorhinal cortex (bilateral; blue), hippocampus (bilateral; medium blue), amygdala (bilateral; light blue), middle frontal gyrus (turquoise), superior/middle temporal gyrus (greenish turquoise), inferior parietal lobule (light green), occipital cortex (green), anterior cingulate (lime), medial temporal cortex (BA37; yellow), inferolateral temporal cortex (bilateral; orange), parieto-occipital junction (dark orange), precuneus (orange-red), premotor cortex (bright red), anterior frontal cortex (red), orbitofrontal cortex (darker red), primary motor cortex (burgundy), choroid plexus (burgundy-brown).

**Supplementary Figure 2**

Results from Figure 1 in the main manuscript but with amount of pathology derived using Markov random fields segmentation. Correlations between PET SUVR and % AT8 positive area in neuropathology images in corresponding cortical brain regions. SUVR = Standardized Uptake Value Ratio. Neuritic plaque score according to NIA-AA [1], grey cross = 0, cyan square = 1, orange cross-circle = 2, blue circle = 3.

**Supplementary Figure 3**


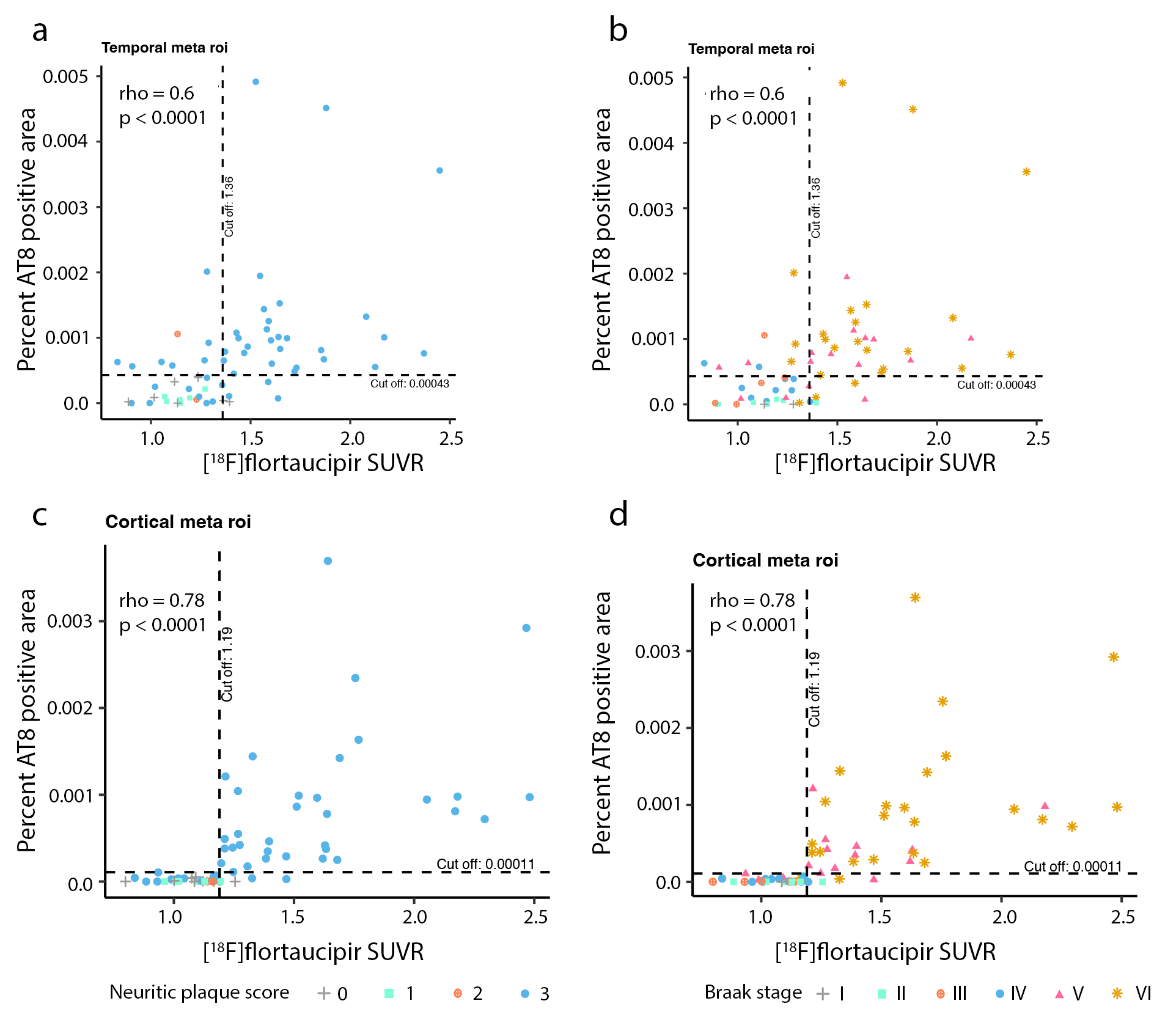


Results from Figure 2 in the main manuscript/Supplementary Figure 3 but with amount of pathology derived using Markov random fields segmentation. Correlation between %AT8 positive area in neuropathology images and [^18^F]flortaucipir SUVR in composite cortical regions. Correlation between PET and pathology in a temporal meta-ROI a-b) with additional visualization of a) the neuritic plaque score and b) Braak stages. In c-d) the correlation in a cortical meta-ROI is shown with visualization of c) the neuritic plaque scores and d) Braak stages. The dashed vertical line indicates the cut-off for [^18^F]flortaucipir SUVR positivity (1.36 in the temporal meta-ROI; 1.19 in cortical composite ROI). The dashed horizontal line represents the cut-off for AT8 positivity at 0.00034 for the temporal meta-ROI (a-b) and 0.00011 for the larger cortical ROI (c-d). a) and c) Neuritic plaque score according to NIA-AA [1], grey cross = 0, cyan square = 1, orange cross-circle = 2, blue circle = 3 [1]. b) and d) Braak stages: grey cross = I, cyan square = II, orange cross-circle = III, blue circle = IV, red triangle = V, golden star = VI. The temporal meta-ROI is defined by an average of left and right entorhinal, superior and medial temporal gyrus, medial temporal (BA37) and left and right inferolateral temporal ROIs. The cortical meta-ROI is defined by an average of ROIs: left middle frontal gyrus, superior and middle temporal gyrus, inferior parietal lobule, occipital cortex (BA 17 and 18), anterior cingulate cortex, medial temporal (BA37), inferolateral temporal left and right, parieto-occipital junction (BA 39), precuneus (BA 7), frontal pre-motor (BA 6), frontal anterior cortex (BA9), orbito-frontal cortex (BA11) and primary motor cortex (BA4). ROI = Region-of-interest.

**Supplementary Figure 4**

**
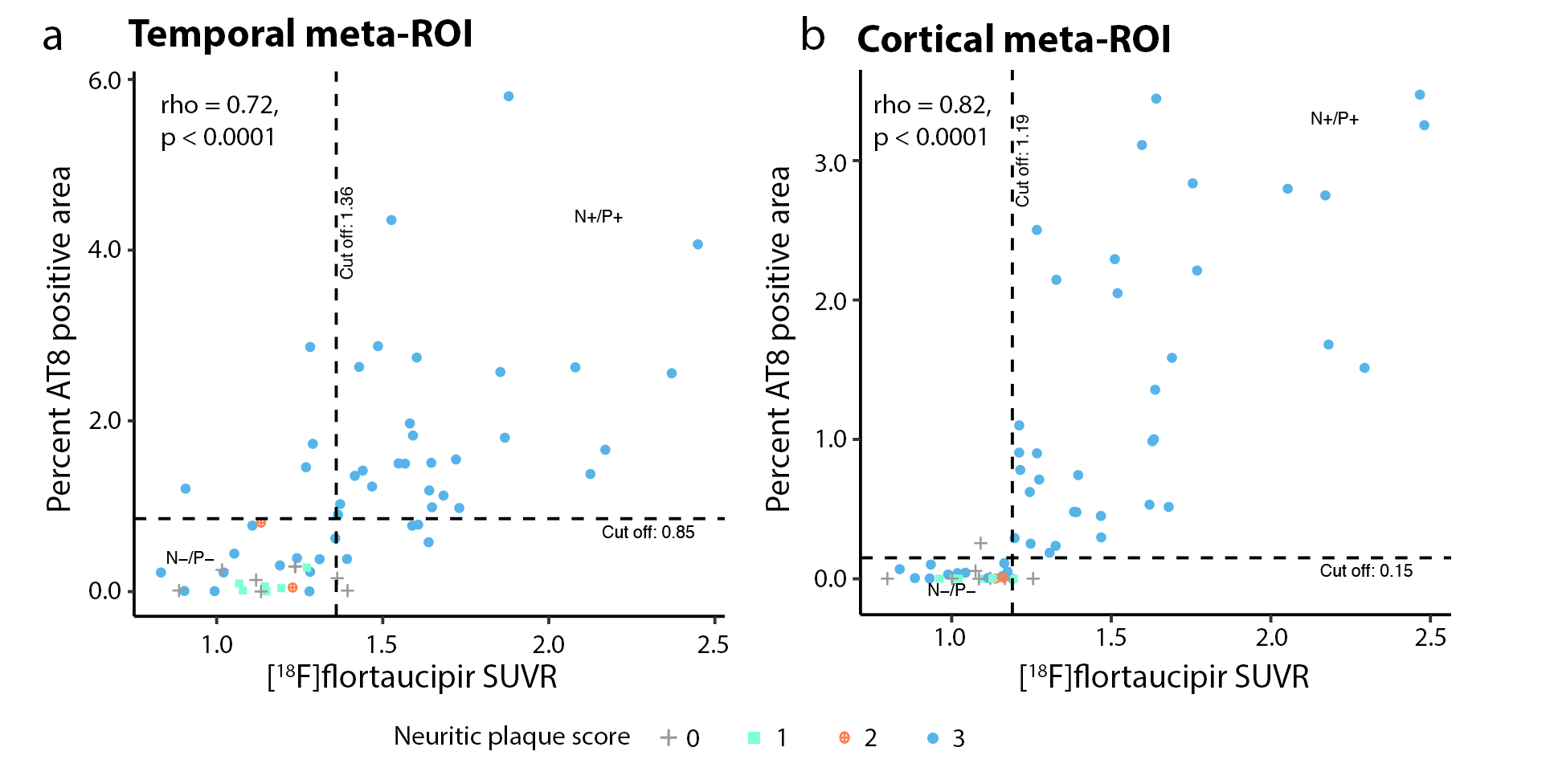
**

Correlation between PET and pathology in a temporal meta-ROI a) and a cortical meta-ROI b) with additional visualization of neuritic plaque scores. The dashed vertical line indicates the cut-off for flortaucipir SUVR positivity (1.36 in the temporal meta-ROI; 1.19 in cortical composite ROI). The dashed horizontal line represents the cut-off for AT8 positivity at 0.85% for the temporal meta-ROI (a) and 0.15% for the larger cortical ROI (b). N-/P-: tau-negativity both in neuropathology and pet. N+/P+: tau-positivity both in neuropathology and pet. Neuritic plaque score according to NIA-AA [1], grey cross = 0, cyan square = 1, orange cross-circle = 2, blue circle = 3. The temporal meta-ROI is defined by an average of left and right entorhinal, superior and medial temporal gyrus, medial temporal (BA37) and left and right inferolateral temporal ROIs. The cortical meta-ROI is defined by an average of ROIs: left middle frontal gyrus, superior and middle temporal gyrus, inferior parietal lobule, occipital cortex (BA 17 and 18), anterior cingulate cortex, medial temporal (BA37), inferolateral temporal left and right, parieto-occipital junction (BA 39), precuneus (BA 7), frontal pre-motor (BA 6), frontal anterior cortex (BA9), orbito-frontal cortex (BA11) and primary motor cortex (BA4). ROI = Region-of-interest.

**Supplementary Figure 5**

**
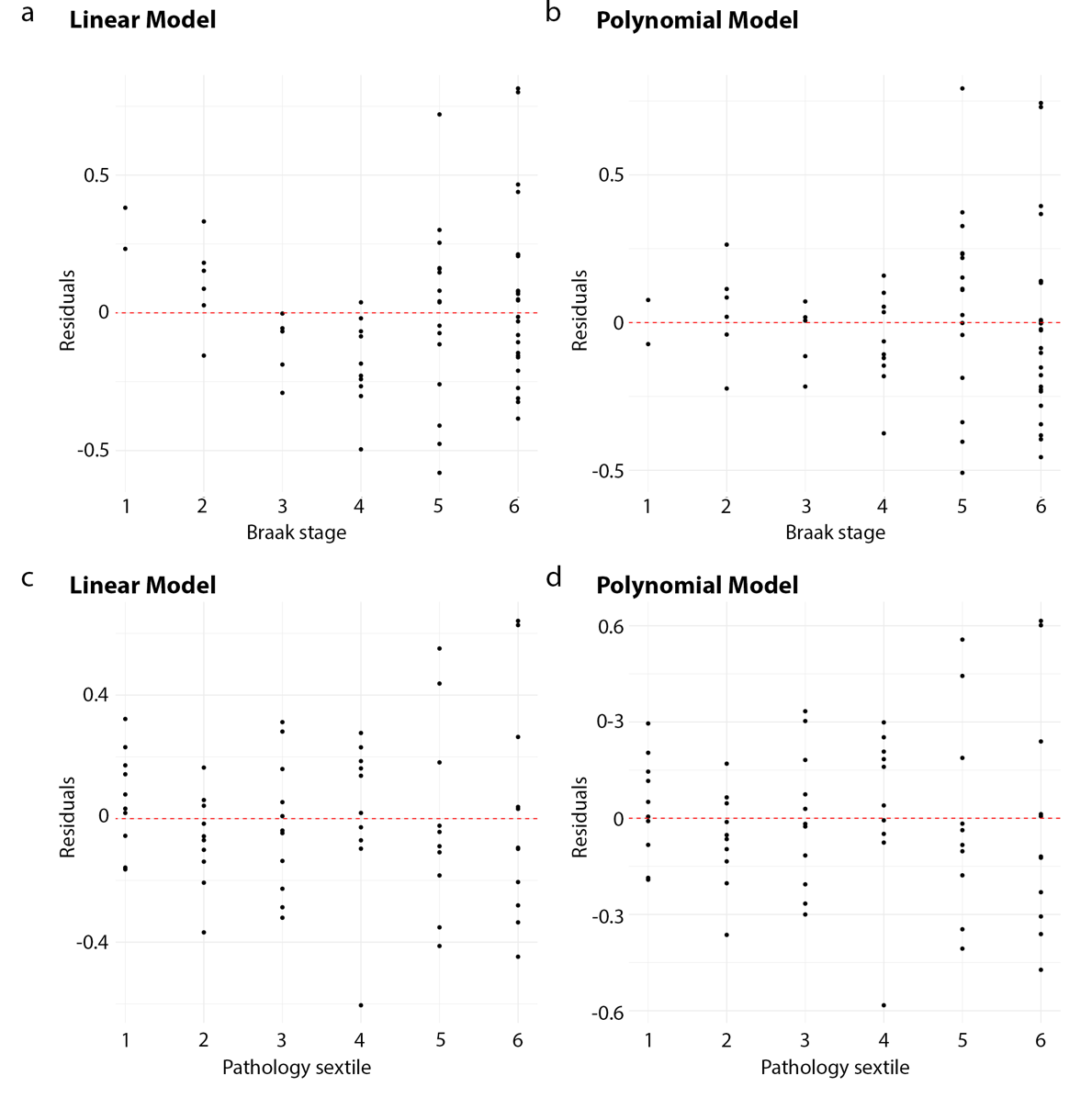
**

|  | **Linear model** | | **Polynomial model** | |  | |
| --- | --- | --- | --- | --- | --- | --- |
|  | **Residual sum of squares** | **AIC** | **Residual sum of squares** | **AIC** | **RSS F-stat p-value** | **ΔAIC** |
| **Braak stages** | 5.09382 | 26.33453 | 4.501038 | 20.54017 | 0.00666 | -5.794 |
| **Pathology sextiles** | 3.783071 | 8.559279 | 3.758574 | 10.1565 | 0.53757 | +1.597 |

Residuals for linear and polynomial models of PET data plotted according to Braak stages (panel a and b) and pathology divided into sextiles (panel c and d). Residuals for linear models are shown in a) and c) and polynomial models in b) and d). The original comparisons are shown in the main manuscript, Figure 4 f and g. Residual sum of squares and Akaike information criterion (AIC) values for the different models as well as the statistical comparisons are shown in the table.

**Supplementary Figure 6**

Correlations of [^18^F]flortaucipir PET retention and neuropathological (AT8) tau in the entorhinal cortex and the amygdala in individuals with low Amyloid-β burden, defined as Thal phases 0 (definite PART), 1 or 2 (possible PART) as indicated. SUVR – standardized uptake value ratio. Thal phase: grey cross = 0, cyan square = 1, orange cross-circle = 2.

**Supplementary Table 2**

Characteristics of the participants with possible or probable PART

| N | 8 |
| --- | --- |
| Age, years (SD)^α^ | 79 (15.8) |
| Sex, female^β^ | 4 (50%) |
| PET to post mortem interval, months (SD) ^α^ | 3.9 (3.1) |
| Thal^β^  0  1  2  3  4  5 | 4 (50%)  2 (25%)  2 (25%)  0 (0%)  0 (0%)  0 (0%) |
| Braak stage^β^  I  II  III  IV  V  VI | 1 (12.5%)  2 (25%)  3 (37.5%)  2 (25%)  0 (0%)  0 (0%) |
| Neuritic plaque score^β^  0  1  2  3 | 6 (75%)  2 (25%)  0 (0%)  0 (0%) |
| ADNC^β^  0  1  2  3 | 4 (50%)  4 (50%)  0 (0%)  0 (0%) |

^α^ Mean (standard deviation). ^β^ *N* (%).

**Supplementary Figure 7**

Correlations between regional neuritic plaque scores and regional [^18^F]flortaucipir SUVR in two extratemporal regions in participants with Braak stages ≤ IV. SUVR = Standardized Uptake Value Ratio. Neuritic plaque score according to NIA-AA [1], grey cross = 0, cyan square = 1, orange cross-circle = 2, blue circle = 3.

**Supplementary Figure 8**


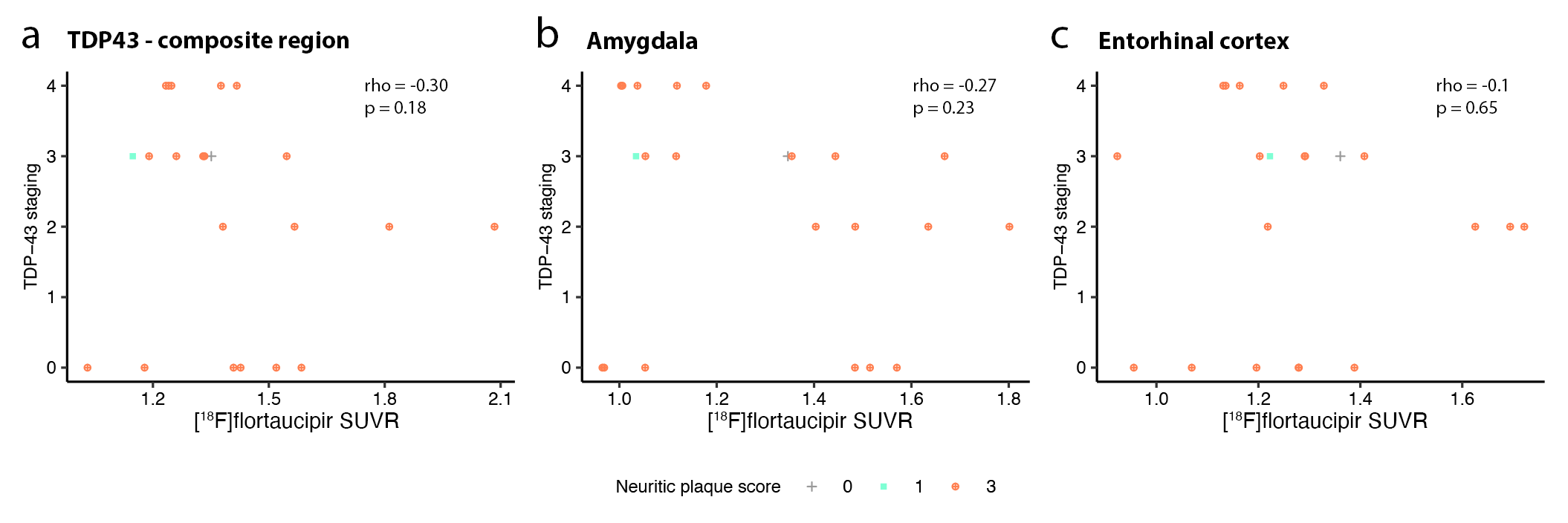


Correlation of TDP-43-stage and [^18^F]flortaucipir SUVRs. a) Composite of amygdala (left), entorhinal cortex (left), orbitofrontal cortex (left) and inferolateral temporal cortex (left). b) Amygdala (left). c) Entorhinal cortex (left). Neuritic plaque score according to NIA-AA [1], grey cross = 0, cyan square = 1, orange cross-circle = 3.

**Supplementary Table 3**

Characteristics of the study participants positive for TDP-43

| N | 22 |
| --- | --- |
| Age, years (SD)^α^ | 86.0 (17.9) |
| Sex, female^β^ | 11 (50%) |
| PET to post mortem interval, months (SD) ^α^ | 2.6 (2.5) |
| Thal^β^  0  1  2  3  4  5 | 1 (4.5%)  1 (4.5%)  0 (0%)  0 (0%)  2 (9.1%)  18 (81.8%) |
| Braak stage^β^  I  II  III  IV  V  VI | 0 (0%)  0 (0%)  0 (0%)  3 (13.6%)  7 (31.8%)  12 (54.5%) |
| Neuritic plaque score^β^  0  1  2  3 | 1 (4.5%)  1 (4.5%)  0 (0%)  20 (90.9%) |
| ADNC^β^  0  1  2  3 | 1 (4.5%)  1 (4.5%)  1 (4.5%)  19 (86.4%) |

^α^ Mean (standard deviation). ^β^ *N* (%).

**Supplementary Figure 9** – PET to neuropathology correlations within pathology and PET positive subjects.

The left panel shows the correlation in participants positive for both neuropathology (N+) and PET (P+), middle panel participants positive for neuropathology (N+) and rightmost panel participants positive in PET (P+). All correlations are significant.

**Supplementary Table 4**

| **[^18^F]Flortaucipir SUVR** | **Detection threshold for tau** |
| --- | --- |
| Temporal meta-ROI | 0.85% |
| Cortical meta-ROI | 0.15% |
| Entorhinal cortex and amygdala (bilateral) | 0.65% |
| **Visual read** | **Detection threshold for tau** |
| Temporal meta-ROI | 0.27% |
| Cortical meta-ROI | 0.11% |
| Entorhinal cortex and amygdala (bilateral) | 0.43% |

A generalized linear model was used to determine threshold for detection of tau pathology using [^18^F]flortaucipir SUVR or visual read. The cut-off for [^18^F]flortaucipir SUVR positivity in both temporal meta-ROI as well as entorhinal cortex and amygdala combined was set to 1.36, the cut-off for [^18^F]flortaucipir SUVR positivity was set to 1.19 [2]. Visual reads were perfomed according to a published visual read algorithm (see [3] for details). SUVR = Standardized Uptake Value Ratio. ROI = Region of Interest.

**Supplementary Figure 10** – A representative example of the ERC ROI localized on an individual [^18^F]flortaucipir PET image.


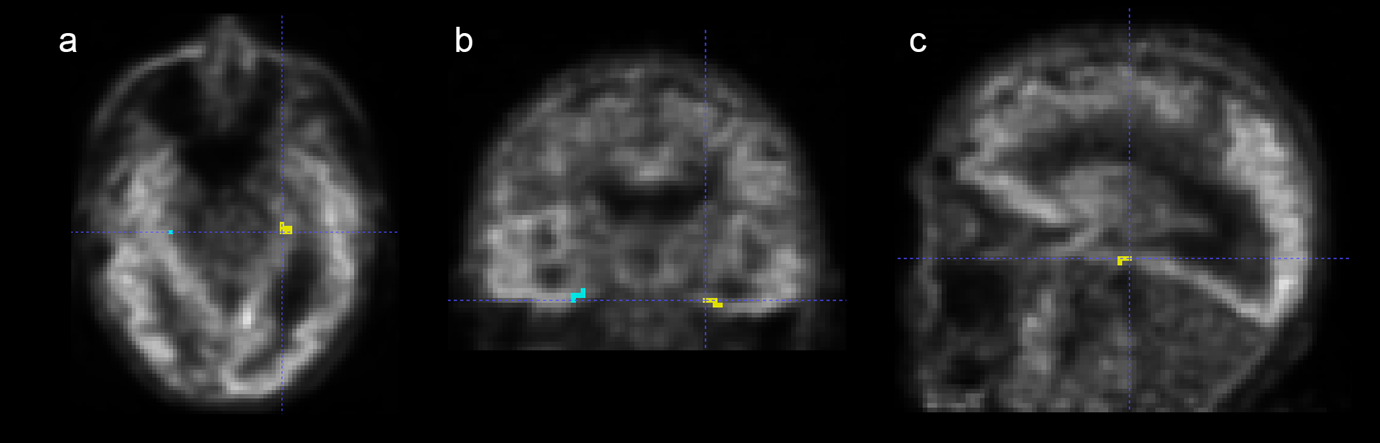


A greyscale [^18^F]flortaucipir image in the a) transversal, b) coronal and c) sagittal plane with a superimposed ROI for the left entorhinal cortex. Parts of the right sided ERC ROI (in turquoise) is visible in two of the planes. The ERC ROI only covers part of the ERC and location was chosen to as closely as possible match the region of neuropathological dissection of the entorhinal cortex.

**References**

1. Hyman BT, Phelps CH, Beach TG, Bigio EH, Cairns NJ, Carrillo MC, Dickson DW, Duyckaerts C, Frosch MP, Masliah E, Mirra SS, Nelson PT, Schneider JA, Thal DR, Thies B, Trojanowski JQ, Vinters H V., Montine TJ (2012) National Institute on Aging-Alzheimer’s Association guidelines for the neuropathologic assessment of Alzheimer’s disease. Alzheimer’s and Dementia 8:1–13. doi: 10.1016/j.jalz.2011.10.007

2. Leuzy A, Pascoal TA, Strandberg O, Insel P, Smith R, Mattsson-Carlgren N, Benedet AL, Cho H, Lyoo CH, Renaud &, Joie L, Rabinovici GD, Ossenkoppele R, Rosa-Neto P, Hansson O (2021) A multicenter comparison of [ 18 F]flortaucipir, [ 18 F]RO948, and [ 18 F] MK6240 tau PET tracers to detect a common target ROI for differential diagnosis. Eur J Nucl Med Mol Imaging 48:2295–2305. doi: 10.1007/s00259-021-05401-4

3.  Smith R, Hägerström D, Pawlik D, Klein G, Jögi , Ohlsson T, Stomrud E, Hansson O (2023) Clinical Utility of Tau Positron Emission Tomography in the Diagnostic Workup of Patients With Cognitive Symptoms. JAMA Neurol. 80(7):749-756. doi: 10.1001/jamaneurol.2023.1323.
